# Tetanus1Read before the Veterinary Medical Association of New Jersey, Atlantic City, September 13, 1900.

**Published:** 1900-12

**Authors:** E. Mathews

**Affiliations:** Jersey City, N. J.


### TETANUS.[1]

By E. Mathews, V.S.,
JERSEY CITY, N. J.

We will not enter into any discussion as to the different divisions given to it by different writers, except to say that it is a continuous contraction of the muscles, causing rigidity of the parts they supply.

[1] Read before the Veterinary Medical Association of New Jersey, Atlantic City, September 13, 1900.

We will speak of tetanus as we find it in our everyday practice. We are called in to see our patient in a more or less advanced stage of sickness, and are told he will not or cannot eat, appears more or less stiff in his gait, and has been so for a day or two. After a more or less careful examination it is pronounced a case of tetanus; prognosis very unfavorable.

As every practitioner is left to his own judgment in the treatment of tetanus, I have tried some experiments on my own account, and will give you the results of some of them.

Case I.—A roan gelding, about five years old, weight about 1200 pounds. I was called to see my patient, November 24, 1897, and was given the privilege of taking him and doing what I saw fit with him. Through the kindness of Dr. G. W. C. Phillips, druggist, I procured a small vial of woorara, having the seal and stamp of the government still unbroken; it was very old. I began by injecting hypodermatically one-eighth of a grain three times a day, increasing it to three-eighths of a grain three times a day.

I had this case under treatment about seventeen days, with favorable results. He is still working. It was a very severe case, his jaws becoming locked very early; at no time did the muscles of respiration become very much affected. I am of the opinion that tetanus in this case was induced by the removal of a tumor about October 1st, the wound to all appearances making a complete recovery.

Case II.—Brown gelding, eight years old, weight about 1500 pounds. Was treated a few months later from same vial of woorara, beginning with two-eighths of a grain three times a day, with favorable results. This case was not as bad as Case I., but was well defined, and was under treatment about ten days. This case used up that vial of woorara. From that time on my success with the drug has been negative. I attribute it to the inferiority of the preparation. I am of the opinion that if we could get a really good article, and could see our patient in the first stage of the disease, we could cure a large percentage.

Having read in some medical journal of the treatment of tetanus with carbolic acid and belladonna, I tried the combination in two cases with favorable results.

Case III.—Bay gelding, six years old, weight about 1450 pounds; in good flesh. Was called in to see him in early summer. When I first saw him tetanus had already developed, yet he had very slight symptoms. I put him under the carbolic acid and belladonna treatment. He was out of harness about five weeks. Is at present working in a lumber truck all right. I gave him about thirty drops each in a little water with a hard-rubber syringe.

CASE IV.—Brown gelding, about twelve years old, used as a driving horse; very high-strung and nervous; weight about 1100 pounds.

I was called in about May 1st. A well-developed case from the start. Had the patient taken to another part of the stable in a narrow stall, darkened. Gave him same doses as Case III. Had him under treatment about two months, greater part of the time in a sling. In all of the cases I kept some thin gruel before them.

In this case I had a very careful, painstaking nurse, one who did just as he was told, which is a great help to us. These are the only cases which I have treated with this combination, and am well pleased with the results.

